# Identification of novel mutations in Chinese Hans with autosomal dominant polycystic kidney disease

**DOI:** 10.1186/1471-2350-12-164

**Published:** 2011-12-20

**Authors:** Chaowen Yu, Yuan Yang, Lin Zou, Zhangxue Hu, Jing Li, Yunqiang Liu, Yongxin Ma, Mingyi Ma, Dan Su, Sizhong Zhang

**Affiliations:** 1 Department of Medical Genetics, and State Key Laboratory of Biotherapy, West China Hospital, Sichuan University, Chengdu, Sichuan, 610041, P. R. China; 2 Department of Nephrology, West China Hospital, Sichuan University, Chengdu, Sichuan, 610041, P. R. China; 3 Center for Clinical Molecular Medicine, Children's Hospital, Chongqing Medical University, Chongqing, 400014, P. R. China

## Abstract

**Background:**

Autosomal dominant polycystic kidney disease (ADPKD) is the most common inherited renal disease with an incidence of 1 in 400 to 1000. The disease is genetically heterogeneous, with two genes identified: *PKD1 *(16p13.3) and *PKD2 *(4q21). Molecular diagnosis of the disease in at-risk individuals is complicated due to the structural complexity of *PKD1 *gene and the high diversity of the mutations. This study is the first systematic ADPKD mutation analysis of both *PKD1 *and *PKD2 *genes in Chinese patients using denaturing high-performance liquid chromatography (DHPLC).

**Methods:**

Both *PKD1 *and *PKD2 *genes were mutation screened in each proband from 65 families using DHPLC followed by DNA sequencing. Novel variations found in the probands were checked in their family members available and 100 unrelated normal controls. Then the pathogenic potential of the variations of unknown significance was examined by evolutionary comparison, effects of amino acid substitutions on protein structure, and effects of splice site alterations using online mutation prediction resources.

**Results:**

A total of 92 variations were identified, including 27 reported previously. Definitely pathogenic mutations (ten frameshift, ten nonsense, two splicing defects and one duplication) were identified in 28 families, and probably pathogenic mutations were found in an additional six families, giving a total detection level of 52.3% (34/65). About 69% (20/29) of the mutations are first reported with a recurrent mutation rate of 31%.

**Conclusions:**

Mutation study of *PKD1 *and *PKD2 *genes in Chinese Hans with ADPKD may contribute to a better understanding of the genetic diversity between different ethnic groups and enrich the mutation database. Besides, evaluating the pathogenic potential of novel variations should also facilitate the clinical diagnosis and genetic counseling of the disease.

## Background

Autosomal dominant polycystic kidney disease (ADPKD) is a severe inherited disorder accounting for up to 10% of end-stage renal diseases [1]. The disease is characterized by numerous gradually enlarging fluid-filled epithelial cysts in bilateral kidneys. Two mapped genes, *PKD1 *(MIM 601313) and *PKD2 *(MIM 173910), are known to cause the disease [2,3]. The former, mutated in 85% of all cases, encodes polycystin-1 (PC1), which is a receptor protein for cell-cell/matrix interactions in the regulation of cell proliferation and apoptosis; the latter, mutated in 15% of the cases, encodes polycystin-2 (PC2), which functions as a transient receptor potential ion channel and regulates intracellular Ca^2+ ^concentration. PC1 interacts with PC2 to form a functional complex that acts as a flow-dependent mechanosensor for regulating the differentiated state of tubular epithelial cells [4-7].

Early diagnosis of ADPKD is established primarily by ultrasound imaging with age-related cyst number criteria [8], however, for younger at-risk individuals and those with *PKD2 *mutations, ultrasonography may be insufficient for providing a definite diagnosis [9,10]. In such cases, linkage analysis is helpful for predicting the diagnosis, but it requires the participation of at least two affected relatives. In recent years, mutation screening, a direct and efficient approach, has been proven applicable to all cases suspected of ADPKD. According to a recent study, the mutation detection rate is approximately 86% using a combination of SURVEYOR Nuclease-Wave HS analysis and direct sequencing [11]. However, mutation screening of *PKD *genes for clinical diagnostic purposes has been proven difficult because of the structural complexity of *PKD *genes and the high diversity of their mutations.

The *PKD1 *gene encodes an approximately 14 Kb transcript with 46 exons extending to 50 kb of the genomic DNA [12] and the 5' part of this gene covering exons 1-33 is duplicated three or more times proximally on chromosome 16 [13]. Therefore, locus-specific amplification of *PKD1 *is required to acquire a single copy of the gene's duplicated region. *PKD2*, which encodes a 3 kb open reading frame with 15 exons, extends to a 70 kb genomic area [14]. Furthermore, no hot mutation in both genes has been reported, and distinguishing the pathogenic mutations from non-pathogenic variations remains a major difficulty in direct gene diagnosis of the disease.

In the present study, a group of novel mutations discovered from the direct mutation screening of both *PKD1 *and *PKD2 *in 65 Chinese families with ADPKD were described. A total of 100 unrelated normal controls were also recruited to differentiate between possible mutations and polymorphic changes. All mutation data detected would be helpful in direct gene diagnosis, as well as in genetic counseling in clinical practice.

## Methods

### The patients and the normal controls

A total of 121 individuals from 65 unrelated families were recruited from West China Hospital, Sichuan University. Among them, 86 individuals were diagnosed with ADPKD according to the ultrasound criteria recommended by Ravine et al. The general clinical data of the patient cohort are summarized in Table 1. In addition, 100 unrelated healthy volunteers 35 to 56 years old were also recruited as controls after exclusion of any renal cysts by ultrasound examination. Peripheral blood samples were collected from all participants, with prior informed consent. This study was approved by the Institutional Ethical Review Boards, Sichuan University.

**Table 1:** General clinical data of the studied patients

General description	Rate (N = 65)^a^
%Female	49.3%
Average age at the time of test (years)	47.2 (range, 28-79)
%ESRD^b^	29.8%
Average GFR (mL/min)^c^	41.4 (range, 3.4-105.1)
Average Serum creatinine concentration (μmol/L)	310.8 (range, 52.0-1022.8)
Average BUN (mmol/L) ^c^	12.3 (range, 3.4-41.8)
%With family history^d^	72.6%

^a ^Data summarized from 65 probands.

^b ^ESRD defined as transplant, dialysis, or Modification of Diet in Renal Disease (MDRD) GER < 10 mL/min.

^c ^MDRD GFR = 186 × (Scr/88.4)^-1.154 ^× Age^-0.203 ^× (0.742 if female). Scr, Serum Creatinine in μmol/L; BUN, blood urea nitrogen in mmol/L.

^d ^Data obtained from the probands and their living family members.

### PCR amplification

For each proband, the genomic DNA was extracted from the peripheral blood sample using a standard phenol-chloroform procedure and prepared in duplicate. The duplicated region of *PKD1 *encoding exons 1-33 was amplified as five specific long fragments, and electrophoresed on 1% agarose gel to detect the possible large range of sequence rearrangement [15-17]. Then, the five long-range PCR products were diluted 1:10^4 ^to avoid genomic DNA carryover, and served as templates for 50 nested PCR reactions. Meanwhile, exons from the unique region of *PKD1 *gene and the entire *PKD2 *gene were amplified from the genomic DNA by 31 additional PCR reactions. A total of 81 PCR products, ranging from 150 to 450 bp, were separated on 2.0% agarose gel to check the amplification efficiency, then were prepared for DHPLC analysis.

### Mutation screening by DHPLC

DNA fragments were analyzed using an automated WAVE Nucleic Acid Fragment Analysis System (Transgenomic, Omaha, Nebraska). Wavemaker 4.2 software (Transgenomic, Omaha, Nebraska) was used to determine the optimal melting temperature for the tested fragments. Heteroduplexes of amplicons were generated prior to DHPLC by denaturing the PCR products at 94°C for 5 min and cooling them at room temperature for 45 min. Then, about 5-8 μL of each product was injected into a high-throughput DNASep column and eluted with a linear acetonitrile gradient of 2% per minute at a flow rate of 0.9 mL min^-1^. All chromatograms were grouped based on the differences in profiles between the normal controls and patients.

### DNA sequencing

All fragments with aberrant elution profiles were sequenced to confirm the possible changes using the same forward and reverse PCR primers used for the PCR amplifications. The change was checked with the duplicate sample after it was found. A necessary cloning and sequencing of the fragment was performed if more than one sequence changes were observed in a DNA fragment. The NCBI RefSeq sequences were used for *PKD1 *[GeneBank: NM_001009944.2] and *PKD2 * in this study. The standard nomenclature recommended by HGVS was used to number nucleotides and name mutations or variations [18,19].

### Evaluation of the pathogenicity of sequence variations

Frame-shifting deletions or insertions, nonsense, typical splicing and in-frame changes of five or more amino acids were defined as pathogenic mutations in this study [20]. Pathogenic potential of missense, intronic changes and synonymous were evaluated using a method recommended by Tan et al.

Firstly, gene variations were classified by analyzing recurrence as reported in the literature, ADPKD mutation database (PKDB) [21] and Single Nucleotide Polymorphism database (dbSNP). Secondly, novel variations and previous unclassified variations were checked in family members available and 100 unrelated normal controls. Variations found in unaffected family members or unrelated normal controls were classified as polymorphisms. Then, the functional significance of the remaining unclassified variations was evaluated computationally using web-based software programs. SIFT and PolyPhen-2 were used to predict possible impact of substitutions on protein function and/or structure [22-26]. The Align-GVGD program was used to determine the Grantham Matrix Score (GMS) for evaluating evolutionary conservation (Grantham Variation[GV]) and chemical differences of resulting amino acid substitutions (the Grantham Distance[GD]) [27-29]. Potential splice-site effects were predicted using NNSplice and NetGene2 with default settings for missense, synonymous, and intronic changes [30-34].

All variations analyzed by these web-based software programs were finally sorted into four categories: 1) probable pathogenic; 2) indeterminate; 3) probable polymorphism; and 4) polymorphism. Only gene variations that were unanimously predicted to be deleterious by SIFT, PolyPhen-2 and Align-GVGD or to affect splicing by NNSplice and NetGene2 were considered to be "probably pathogenic", if no other definite mutation was found in the same patient. If a definite mutation coexisted with a deleterious missense change or a likely atypical splicing variation in the same patient, the missense change and the atypical splicing variation were considered to be "indeterminate". Similarly, only variations that were scored as begin or predicted to have no effect on splicing by all corresponding applications were considered to be "polymorphisms". Otherwise, they were classified as "probable polymorphisms".

## Results

In total, 92 different gene variations were detected. Among them, 23 pathogenic mutations and 6 probably pathogenic mutations, with 26 located in *PKD1 *and 3 in *PKD2*, were found in 34 families (Table 2), giving a mutation detection rate of 52.3% (34/65). Novel mutations were found in 69% (20/29) of the mutations with a recurrent rate of 31% (9/29). The most common mutation, NM_001009944.2: c.5014_5015delAG, was found in three families. Two nonsense mutations (NP_001009944.2: p.Tyr2796* and NP_000288.1: p.Arg325*), one deletion (NP_001009944.2: p.Asn2925Tyrfs*10) were found twice.

**Table 2:** Characteristics of the detected mutations

Description	*PKD1*	*PKD2*	Total
Pathogenic	21	3	23
Probably pathogenic	5	1	6
FS deletion/insertion/duplication	10	0	10
Nonsense	9	1	10
Splicing	1	1	2
IF deletion/insertion	1	0	1
Missense	5	1	6
Recurrent mutations	7	2	9 (31%)
Novel mutations	19	1	20 (69%)
Total mutations detected	26 (89.7%)	3 (10.3%)	29

FS, frame-shift; IF, in-frame.

Definite mutations were found in 28 of the families including 10 frameshift, 10 nonsense, two typical splicing and one duplication of five amino acids. These disease-causing mutations are reported in Table 3. Totally 28 missense changes were detected in the patients, of which 9 were reported as polymorphisms previously. Additionally, NP_001009944.2: p.Ser372Asn and p.Arg2654Gly that coexisted with a definite mutation NP_001009944.2: p.Arg2430* in patient 09032 were found in unaffected family members; NP_001009944.2: p.Leu1290Val that coexisted with NP_001009944.2: p.Arg462fs in patient 08006, NP_001009944.2: p.Arg3169Gln that coexisted with NP_001009944.2: p.Trp3785* in patient 08020, and NP_001009944.2: p.Ala1792Thr in patient 09026 were found in unrelated normal controls; these five missense variations were classified as polymorphisms. The pathogenic potential of the remaining 14 unclassified missense changes were evaluated by SIFT, PolyPhen-2 and Align-GVGD (see Additional file 1). Finally, additional six were predicted to be deleterious by all three software applications, and classified as "probably pathogenic" (Table 4); two were scored as benign unanimously and defined as "polymorphisms"; others scored as deleterious by only one or two of these applications were considered to be "probable polymorphisms".

**Table 3:** Details of pathogenic mutations observed from *PKD1 *and *PKD2 *

Patient ID	Region	cdna Change	Amino Acid Change	Type	Previous description
*PKD1*					
08006	IVS7	c.1606+1G > A	p.Arg462fs	Splice	PD
09065	EX9A	c.1779delA	p.Glu593Aspfs*192	Frameshift	Novel
09030	EX13	c.3058C > T	p.Gln1020*	Nonsense	PD
09041	EX15B	c.3824delG	p.Gly1275Valfs*71	Frameshift	Novel
09052	EX15E	c.4746G > A	p.Trp1582*	Nonsense	PD
08011	EX15F	c.5014_5015delAG	p.Arg1672Glyfs*98	Frameshift	PD
08019	EX15F	c.5014_5015delAG	p.Arg1672Glyfs*98	Frameshift	PD
09034	EX15F	c.5014_5015delAG	p.Arg1672Glyfs*98	Frameshift	PD
09060	EX15H	c.5595delG	p.Leu1866Serfs*83	Frameshift	Novel
09056	EX15H	c.5722C > T	p.Gln1908*	Nonsense	Novel
08013	EX15M	c.6424C > T	p.Gln2142*	Nonsense	Novel
09024	EX15N	c.6650_6664dup15	p.Val2217_Leu2221dup	Duplication	Novel
09069	EX15N	c.6730_6731delAG	p.Ser2244Hisfs*17	Frameshift	Novel
08008	EX15N	c.6781delG	p.Glu2261Argfs*53	Frameshift	Novel
09032	EX18	c.7288C > T	p.Arg2430*	Nonsense	PD
09031	EX23A	c.8388T > A	p.Tyr2796*	Nonsense	Novel
09042	EX23A	c.8388T > A	p.Tyr2796*	Nonsense	Novel
08023	EX23B	c.8614DelA	p.Ile2872Serfs*3	Frameshift	Novel
08002	EX23B	c.8772_8776delCAACT	p.Asn2925Tyrfs*10	Frameshift	Novel
09066	EX23B	c.8772_8776delCAACT	p.Asn2925Tyrfs*10	Frameshift	Novel
09037	EX29	c.9840_9843dupGGCC	p.Thr3282Glyfs*109	Frameshift	Novel
09035	EX35	c.10527_10528delGA	p.Glu3509Aspfs*117	Frameshift	Novel
08020	EX40	c.11354G > A	p.Trp3785*	Nonsense	Novel
09063	EX44	c.12013C > T	p.Gln4005*	Nonsense	PD
09058	EX44	c.12061C > T	p.Arg4021*	Nonsense	PD
*PKD2*					
09047	EX4	c.973C > T	p.Arg325*	Nonsense	PD
09070	EX4	c.973C > T	p.Arg325*	Nonsense	PD
09036	IVS4	c.1094+1G > C	p.Ala365fs	Splice	PD

PD, previously described in other studies, details in the Human Gene Mutation Database (HGMD) and/or the Autosomal Dominant Polycystic Kidney Disease: Mutation Database (PKDB).

**Table 4:** Details of the probably pathogenic mutations

Patient ID	Region	cdna change	Amino Acid Change	Type	Previous description
*PKD1*					
08012	EX15E	c.4810G > A	p.Val1604Met	Substitution	Novel
09049	EX26	c.9335G > T	p.Cys3112Phe	Substitution	Novel
08009	EX26	c.9388C > T	p.Arg3130Trp	Substitution	Novel
09028	EX29	c.9884A > G	p.Asn3295Ser	Substitution	Novel
09040	EX40	c.11285C > T	p.Pro3762Leu	Substitution	Novel
*PKD2*					
09039	EX4	c.1034A > G	p.Tyr345Cys	Substitution	Novel

Novel synonymous variations, intronic changes, and missense variations scored as "benign" or "unclassified" by SIFT, PolyPhen-2 and Align-GVGD were evaluated for splice-site effects (see Additional file 2). Three variations, NM_001009944.2: c.7704-12C > T, NM_001009944.2: c.7796T > G (p.Leu2599Arg), and NM_001009944.2: c.10618+16_10618+18delinsAAA were predicted to have a slight effect on splice-site by only one of the applications and therefore were considered to be "probable polymorphisms". Based on the analysis criteria indicated above, six variations were predicted to be "probably pathogenic", eight were classified as "probable polymorphisms", and 55 were classified as "polymorphisms". These polymorphisms and probable polymorphisms are shown in Additional file 3.

## Discussion

Mutation analysis of *PKD1 *and *PKD2 *in Chinese ADPKD patients previously focused on the unique region of the genes [35-37], only one systematic mutation analysis of both genes in Chinese patients by single-strand conformation polymorphism (SSCP) has been reported, which contained only 24 families [38]. Therefore, it is essential to understand how mutations are distributed in all regions of the genes in Chinese patients and the genetic diversity between different ethnic groups. The present study has analyzed 65 ADPKD families using DHPLC and DNA sequencing, giving a mutation detection rate of 52.3%. Among the 29 mutations, 69% are reported for the first time, and recurrent mutations account for about 31%. No hot mutation was found in this study.

Gene mutations detected in the study include frameshift, nonsense, missense, and splice-site changes, and the proportion of each type of mutation is in agreement with that reported by Rossetti et al. (P > 0.05, Chi-square Test). In total of 62 variations detected in *PKD1 *gene of the patients, 21 variations were located in exon 15, and accounted for 33.9% (21/62) which is higher than that recorded in PKDB (181/873) (P < 0.05, Chi-square Test). No definitely pathogenic mutation that coexisted with another mutation in a same patient was found in our study of Chinese patients, only some missense, synonymous, or intronic changes were found to coexist with a definite pathogenic mutation, but were scored as benign according to the analysis criteria. A duplication of five amino acids (NP_001009944.2: p.Val2217_Leu2221dup) found in patient 09024 was an exonic rearrangement that may affect the structure of the protein, therefore, was classified as a pathogenic mutation as recommended by Rossetti et al.

A number of sequence changes in the 5' replicated region of *PKD1 *were also present in their homologous locis, such as the mutations NM_001009944.2: c.7288C > T (p.Arg2430*), c.8614DelA (p.Ile2872Serfs*3), and four polymorphisms, NM_001009944.2: c.1849+14_1849+26delTGGTGGGTGGTGG, c.8087T > G (p.Leu2696Arg), c.8681_8689delCCAACTCCG (p.Ala2894_Ser2896del) and c.9506G > A (p.Arg3169Gln). Nucleotide sequences of *PKD1 *exhibiting these changes were identical to at least one of their homologous copies on chromosome 16. A similar phenomenon was also observed in other ethnic groups [39,40], especially in a population of 41 unrelated Thai and six unrelated Korean families with ADPKD by Phakdeekitcharoen et al. A possible reason for the phenomenon is that gene conversion has happened between *PKD1 *and its homologous loci [41,42]. Nevertheless, this kind of variations should be interpreted carefully when utilized in clinical diagnosis.

Because of the high prevalence of polymorphisms and private mutations, particularly in *PKD1*, it is difficult to determine whether a specific genetic change is a mutation or a polymorphism. In the present study, all novel variations and previous unclassified variations were first checked in family members and unrelated normal controls. Then the pathogenic potential of the remaining variations were analyzed by web-based software applications. SIFT, PolyPhen-2, NNSplice, and NetGene2 were used with default setting. The GMS was used to score the GD and GV of each substitution, and this method has been currently automated in the program Align-GVGD. All analyses have finally identified additional six probably pathogenic mutations, increasing the overall detection rate to 52.3%, and this result demonstrates the utility of bioinformatics evaluation of gene variations in *PKD *genes.

DHPLC is a well-known method that could be used for the detection of heterozygous variants. However, DNA fragments with variants located in the GC-rich region or the 5' and 3' ends nearby tend not to generate recognizable elution peaks, which could lead to false negative results [43]. Therefore, direct sequencing both *PKD1 *and *PKD2 *genes of patients, especially the mutation-negative cases, could be one of the most efficient methods for mutation detection. Large deletions and duplications were reported to account for 1%-3% of the mutations in *PKD1 *patients [44,45], however, if the rearrangement extends beyond the limits of the large amplicons, only the wild-type would be amplified by PCR. For this kind of mutations, multiplex ligation-dependent probe amplification may serve as a more reliable detection assay [46,47]. Considering the structural complexity of the *PKD1 *gene and the diversity of mutation types, a combination of multiple methods rather than a single assay is highly recommended to meet the patients' demand for a complete molecular genetic diagnosis of ADPKD.

## Conclusions

The present mutation analysis of *PKD1 *and *PKD2 *genes in Chinese Hans with ADPKD may contribute to a better understanding of the genetic diversity between different ethnic groups and enrich the mutation database. Besides, evaluating the pathogenic potential of novel variations should also facilitate the clinical diagnosis and genetic counseling of the disease, particularly through the direct gene approach.

## Competing interests

The authors declare that they have no competing interests.

## Authors' contributions

CY carried out the molecular genetic studies and drafted the manuscript. YY and SZ designed the experiments and revised this article. ZH and JL provided a part of patient subjects and the ultrasound examination of the patients. LZ, YM, MM, DS and YL analyzed the mutation data and helped to classify all the variations. All authors read and approved the final manuscript.

## Pre-publication history

The pre-publication history for this paper can be accessed here:

http://www.biomedcentral.com/1471-2350/12/164/prepub

## Supplementary Material

Additional file 1 **Supplementary Table S1**. **Evaluation of the Pathogenic Potential of Missense Variants**. The pathogenic potential of missense variants was evaluated by SIFT, PolyPhen-2 and Align-GVGD. For Align-GVGD analysis, an multisequence alignment (MSA) was generated using the software T-coffee http://tcoffee.crg.cat/apps/tcoffee/play?name=regular of *PKD1 *orthologs from human (GI 205360954), monkey (GI 297283254), mouse (GI 124487380), rat (GI 293351352), dog (GI 54792752), chicken (GI 118097923), frog (Xenopus [Silurana] tropicalis GI 301605771), fish (Fugu, ENSTRUP0000002017). For PKD2, human (GI 4505835), monkey (GI 109074954), mouse (GI 164519057), rat (GI 300794239), dog (GI 74002219), chicken (GI 71896749), frog (GI 301618537), fish (Danio rerio GI50539686), cow (GI 114052611), fish (Oryzias latipes GI 211904097), pig (Sus scrofa GI 311262841) were used. Sequences were obtained from NCBI or Ensembl database. We entered the two gene alignments using default settings. This method has been currently automated in the program Align-GVGD. Click here for file

Additional file 2 **Supplementary Table S2. Atypical splicing prediction**. Atypical splicing prediction of novel synonymous variations, intronic changes, and missense variations was performed using NNSplice and NetGene2. Click here for file

Additional file 3 **Supplementary Table S3**. **Summary of *PKD1 *and *PKD2 *Genetic Variations (Polymorphisms, Probable Polymorphisms)**. A brief summary of polymorphisms and probable polymorphisms detected from patients, unaffected family members and normal controls in this study. Click here for file
