# Supplementary material for: Identification of novel mutations in Chinese Hans with autosomal dominant polycystic kidney disease
Source: BMC Med Genet. 2011 Dec 20;12:164. doi: 10.1186/1471-2350-12-164 (PMC3341574; doi:10.1186/1471-2350-12-164)
Supplement: Additional file 1 — Supplementary Table S1. Evaluation of the Pathogenic Potential of Missense Variants. The pathogenic potential of missense variants was evaluated by SIFT, PolyPhen-2 and Align-GVGD. For Align-GVGD analysis, an multisequence alignment (MSA) was generated using the software T-coffee http://tcoffee.crg.cat/apps/tcoffee/play?name=regular of PKD1 orthologs from human (GI 205360954), monkey (GI 297283254), mouse (GI 124487380), rat (GI 293351352), dog (GI 54792752), chicken (GI 118097923), frog (Xenopus [Silurana] tropicalis GI 301605771), fish (Fugu, ENSTRUP0000002017). For PKD2, human (GI 4505835), monkey (GI 109074954), mouse (GI 164519057), rat (GI 300794239), dog (GI 74002219), chicken (GI 71896749), frog (GI 301618537), fish (Danio rerio GI50539686), cow (GI 114052611), fish (Oryzias latipes GI 211904097), pig (Sus scrofa GI 311262841) were used. Sequences were obtained from NCBI or Ensembl database. We entered the two gene alignments using default settings. This method has been currently automated in the program Align-GVGD. [file 1471-2350-12-164-S1.DOC]

Supplementary Table 1. Evaluation of the Pathogenic Potential of Missense Variants

| Region | Substitution | SIFT | |  | PolyPhen-2 | |  | Align-GVGD | | |
| --- | --- | --- | --- | --- | --- | --- | --- | --- | --- | --- |
|  |  | Score | Prediction |  | Score | Prediction |  | GV | GD | Prediction |
| *PKD1* |  |  |  |  |  |  |  |  |  |  |
| EX5C | p.Ser372Asn | 0.01 | DAMAGING |  | 0.135 | BENIGN |  | 215.31 | 38.84 | Neutral |
| EX10A | p.Ser629Thr | 0.34 | TOLERATED |  | 0.001 | BENIGN |  | 111.88 | 0 | Neutral |
| EX11A | p.Arg799Gln | 0.34 | TOLERATED |  | 0.996 | DAMAGING |  | 121.10 | 12.36 | Neutral |
| EX11B | p.Arg832Gly | 0.13 | TOLERATED |  | 0.014 | BENIGN |  | 116.23 | 83.08 | Unclassified |
| EX15B | p.Leu1290Val | 0.55 | TOLERATED |  | 0.003 | BENIGN |  | 161.57 | 0 | Neutral |
| EX15E | p.Val1604Met | 0.01 | DAMAGING |  | 0.993 | DAMAGING |  | 0 | 20.52 | Deleterious |
| EX15G | p.Ala1792Thr | 0.2 | TOLERATED |  | 0.028 | BENIGN |  | 154.69 | 0 | Neutral |
| EX18 | p.Thr2414Met | 0 | DAMAGING |  | 0.994 | DAMAGING |  | 215.31 | 64.73 | Unclassified |
| EX19 | p.Asp2557Gly | 0 | DAMAGING |  | 0.999 | DAMAGING |  | 213.16 | 64.73 | Unclassified |
| EX20 | p.Leu2599Arg | 0 | DAMAGING |  | 0.998 | DAMAGING |  | 234.72 | 91.58 | Unclassified |
| EX21 | p.Arg2654Gly | 0.43 | TOLERATED |  | 0.983 | DAMAGING |  | 244.67 | 24.28 | Neutral |
| EX23A | p.Gly2798Cys | 0.02 | DAMAGING |  | 0.125 | BENIGN |  | 142.48 | 112.77 | Unclassified |
| EX26 | p.Cys3112Phe | 0 | DAMAGING |  | 0.999 | DAMAGING |  | 0 | 204.39 | Deleterious |
| EX26 | p.Arg3130Trp | 0 | DAMAGING |  | 1 | DAMAGING |  | 0 | 101.29 | Deleterious |
| EX27 | p.Arg3169Gln | 1 | TOLERATED |  | 0.008 | BENIGN |  | 44.91 | 0 | Neutral |
| EX29 | p.Asn3295Ser | 0 | DAMAGING |  | 0.995 | DAMAGING |  | 0 | 46.24 | Deleterious |
| EX34 | p.Glu3479Asp | 1 | TOLERATED |  | 0.001 | BENIGN |  | 240.29 | 0 | Neutral |
| EX40 | p.Pro3762Leu | 0.01 | DAMAGING |  | 0.990 | DAMAGING |  | 37.56 | 76.16 | Deleterious |
| *PKD2* |  |  |  |  |  |  |  |  |  |  |
| EX4 | p.Tyr345Cys | 0 | DAMAGING |  | 0.989 | DAMAGING |  | 0 | 193.72 | Deleterious |

An multisequence alignment (MSA) was generated using the software T-coffee ([http://tcoffee.crg.cat/apps/tcoffee/play?name=regular](../Additional%20file%201.doc)) of *PKD1* orthologs from human (GI 205360954), monkey (GI 297283254), mouse (GI 124487380), rat (GI 293351352), dog (GI 54792752), chicken (GI 118097923), frog (Xenopus [Silurana] tropicalis GI 301605771), fish (Fugu, ENSTRUP0000002017). For PKD2, human (GI 4505835), monkey (GI 109074954), mouse (GI 164519057), rat (GI 300794239), dog (GI 74002219), chicken (GI 71896749), frog (GI 301618537), fish (Danio rerio GI50539686), cow (GI 114052611), fish (Oryzias latipes GI 211904097), pig (Sus scrofa GI 311262841) were used. Sequences were obtained from NCBI or Ensembl database. We entered the two gene alignments using default settings. This method has been currently automated in the program Align-GVGD.
