# Supplementary material for: Identification of novel mutations in Chinese Hans with autosomal dominant polycystic kidney disease
Source: BMC Med Genet. 2011 Dec 20;12:164. doi: 10.1186/1471-2350-12-164 (PMC3341574; doi:10.1186/1471-2350-12-164)
Supplement: Additional file 2 — Supplementary Table S2. Atypical splicing prediction. Atypical splicing prediction of novel synonymous variations, intronic changes, and missense variations was performed using NNSplice and NetGene2. [file 1471-2350-12-164-S2.DOC]

Supplementary Table 2. Atypical splicing prediction

| Region | cDNA change | Amino Acid Change | Mutational type | Splicing Predictions | |
| --- | --- | --- | --- | --- | --- |
|  |  |  |  | NNSplice | NetGene2 |
| *PKD1* |  |  |  |  |  |
| EX5C | c.1104G>A | p.(=) | Synonymous | N/P | N/P |
| EX5C | c.1115G>A | p.Ser372Asn | Substitution | N/P | N/P |
| EX10A | c.1885T>A | p.Ser629Thr | Substitution | N/P | N/P |
| EX11A | c.2396G>A | p.Arg799Gln | Substitution | N/P | N/P |
| EX11B | c.2469G>A | p.(=) | Synonymous | N/P | N/P |
| EX11B | c.2494C>G | p.Arg832Gly | Substitution | N/P | N/P |
| EX15B | c.3868C>G | p.Leu1290Val | Substitution | N/P | N/P |
| EX15E | c.4587C>A | p.(=) | Synonymous | N/P | N/P |
| EX15F | c.4941C>T | p.(=) | Synonymous | N/P | N/P |
| EX15H | c.5598C>T | p.(=) | Synonymous | N/P | N/P |
| EX15N | c.6777C>A | p.(=) | Synonymous | N/P | N/P |
| EX18 | c.7241C>T | p.Thr2414Met | Substitution | N/P | N/P |
| EX19 | c.7670A>G | p.Asp2557Gly | Substitution | N/P | ND 0.34 |
| IVS19 | c.7704-12C>T |  | IVS silent | N/P | Strengthened  (0.56 to 0.57) |
| EX20 | c.7796T>G | p.Leu2599Arg | Substitution | Strengthened  (0.61 to 0.71) |  |
| EX21 | c.7960A>G | p.Arg2654Gly | Substitution | N/P | N/P |
| IVS21 | c.8016+16C>A |  | IVS silent | N/P | N/P |
| EX23A | c.8392G>T | p.Gly2798Cys | Substitution | NA 0.42 | N/P |
| EX34 | c.10437G>C | p.Glu3479Asp | Substitution | N/P | N/P |
| IVS35 | c.10618+16_10618+18 delinsAAA |  | IVS silent | N/P | Weakened  (0.32 to 0.31) |
| EX45 | c.12360G>A | p.(=) | Synonymous | N/P | N/P |
| *PKD2* |  |  |  |  |  |
| IVS4 | c.1095-32A>G |  | IVS silent | N/P | N/P |

NP, none predicted; p.(=), synonymous change at protein level.
