# Supplementary material for: Identification of novel mutations in Chinese Hans with autosomal dominant polycystic kidney disease
Source: BMC Med Genet. 2011 Dec 20;12:164. doi: 10.1186/1471-2350-12-164 (PMC3341574; doi:10.1186/1471-2350-12-164)
Supplement: Additional file 3 — Supplementary Table S3. Summary of PKD1 and PKD2 Genetic Variations (Polymorphisms, Probable Polymorphisms). A brief summary of polymorphisms and probable polymorphisms detected from patients, unaffected family members and normal controls in this study. [file 1471-2350-12-164-S3.DOC]

Supplementary Table 3. Summary of *PKD1* and *PKD2* Genetic Variations (Polymorphisms, Probable Polymorphisms)

| Region | cdna change | Amino Acid Change | type | Study groups | Classification | DbSNP rs# | Previous description |
| --- | --- | --- | --- | --- | --- | --- | --- |
| *PKD1* |  |  |  |  |  |  |  |
| IVS2 | c.288-11G>C |  | IVS silent | NC | PM |  | Novel |
| IVS2 | c.288-32A>G |  | IVS silent | NC | PM |  | Novel |
| EX5C | c.1104G>A | p.(=) | Synonymous | P | PM |  | Novel |
| EX5C | c.1115G>A | p.Ser372Asn | Substitution | P,UF | PM |  | Novel |
| IVS9 | c.1849+14_1849+26del TGGTGGGTGGTGG |  | IVS silent | P,NC | PM |  | Novel |
| IVS9 | c.1849+67T>C |  | IVS silent | P,NC | PM |  | Novel |
| IVS9 | c.1849+91A>G |  | IVS silent | NC | PM |  | Novel |
| EX10A | c.1885T>A | p.Ser629Thr | Substitution | P | PM |  | Novel |
| EX11A | c.2098-10C>T |  | IVS silent | NC | PM |  | Novel |
| EX11A | c.2396G>A | p.Arg799Gln | Substitution | P | PPM |  | Novel |
| EX11B | c.2469G>A | p.(=) | Synonymous | P | PM |  | Novel |
| EX11B | c.2494C>G | p.Arg832Gly | Substitution | P | PPM |  | Novel |
| EX11B | c.2527T>C | p.Ser843Pro | Substitution | NC | PM |  | Novel |
| IVS12 | c.2986-15C>T |  | IVS silent | P,UF | PM |  | PD |
| EX15B | c.3719A>T | p.Asn1240Ile | Substitution | NC | PM |  | Novel |
| EX15B | c.3722T>C | p.Ile1241Thr | Substitution | NC | PM |  | Novel |
| EX15B | c.3868C>G | p.Leu1290Val | Substitution | P,NC | PM |  | Novel |
| EX15D | c.4340C>T | p.Ala1447Val | Substitution | P,NC | PM | rs76981724 | NCBI DbSNP |
| EX15E | c.4587C>A | p.(=) | Synonymous | P | PM |  | Novel |
| EX15E | c.4669C>T | p.Arg1557Cys | Substitution | NC | PM | rs150797875 | NCBI DbSNP |
| EX15E | c.4674G>A | p.(=) | Synonymous | P,NC | PM | rs79884128 | PD |
| EX15E | c.4817C>G | p.Thr1606Ser | Substitution | NC | PM |  | Novel |
| EX15F | c.4941C>T | p.(=) | Synonymous | P | PM |  | Novel |
| EX15G | c.5368G>C | p.Ala1790Pro | Substitution | NC | PM |  | Novel |
| EX15G | c.5374G>A | p.Ala1792Thr | Substitution | P,NC | PM |  | Novel |
| EX15H | c.5598C>T | p.(=) | Synonymous | P | PM |  | Novel |
| EX15I | c.5847C>T | p.(=) | Synonymous | P | PM | rs80111665 | PD |
| EX15L | c.6205G>A | p.Gly2069Ser | Substitution | P | PM | rs148363380 | NCBI DbSNP |
| EX15L | c.6231G>A | p.(=) | Synonymous | NC | PM |  | Novel |
| EX15L | c.6331G>A | p.Glu2111Lys | Substitution | P,NC | PM | rs138672759 | NCBI DbSNP |
| EX15N | c.6777C>A | p.(=) | Synonymous | P | PM |  | Novel |
| EX18 | c.7241C>T | p.Thr2414Met | Substitution | P | PPM |  | Novel |
| EX18 | c.7271C>T | p.Thr2424Met | Substitution | NC | PM |  | Novel |
| EX18 | c.7278T>C | p.(=) | Synonymous | NC | PM | rs2575311 | NCBI DbSNP |
| EX18 | c.7480G>A | p.Glu2494Lys | Substitution | NC | PM |  | Novel |
| EX19 | c.7566C>T | p.(=) | Synonymous | NC | PM |  | Novel |
| EX19 | c.7670A>G | p.Asp2557Gly | Substitution | P | PPM |  | Novel |
| IVS19 | c.7704-12C>T |  | IVS silent | P | PPM |  | Novel |
| EX20 | c.7740C>T | p.(=) | Synonymous | NC | PM |  | Novel |
| EX20 | c.7796T>G | p.Leu2599Arg | Substitution | P | PPM |  | Novel |
| EX21 | c.7866C>T | p.(=) | Synonymous | NC | PM |  | PD^b^ |
| EX21 | c.7913A>G | p.His2638Arg | Substitution | NC | PM | rs9936785 | PD^b^ |
| EX21 | c.7918G>C | p.Ala2640Pro | Substitution | NC | PM |  | Novel |
| EX21 | c.7960A>G | p.Arg2654Gly | Substitution | P,UF | PM |  | Novel |
| IVS21 | c.8016+16C>A |  | IVS silent | P | PM |  | Novel |
| EX22 | c.8087T>G | p.Leu2696Arg | Substitution | P | PM |  | PD |
| EX23A | c.8392G>T | p.Gly2798Cys | Substitution | P | PPM |  | Novel |
| EX23B | c.8444C>T | p.Ala2815Val | Substitution | NC | PM |  | Novel |
| EX23B | c.8681_8689del CCAACTCCG | p.Ala2894_Ser2896del | Deletion | P | PM |  | PD |
| EX25 | c.9156C>T | p.(=) | Synonymous | P | PM |  | PD |
| EX27 | c.9421A>C | p.Met3141Leu | Substitution | NC | PM |  | Novel |
| EX27 | c.9506G>A | p.Arg3169Gln | Substitution | P,NC | PM |  | Novel |
| EX27 | c.9534C>T | p.(=) | Synonymous | NC | PM |  | Novel |
| EX27 | c.9548G>A | p.Arg3183Gln | Substitution | NC | PM | rs79648977 | NCBI DbSNP |
| EX34 | c.10437G>C | p.Glu3479Asp | Substitution | P | PM |  | Novel |
| EX35 | c.10529C>T | p.Thr3510Met | Substitution | P,UF,NC | PM | rs45478794 | PD |
| EX35 | c.10556_10565  delinsAGTT | p.Gly3519_Ser3522  delinsGluPhe | In-frame delins | NC | PM |  | Novel |
| IVS35 | c.10618+16_10618+18 delinsAAA |  | IVS silent | P | PPM |  | Novel |
| EX40 | c.11333C>A | p.Thr3778Asn | Substitution | P | PM | rs114656915 | NCBI DbSNP |
| EX45 | c.12360G>A | p.(=) | Synonymous | P | PM |  | Novel |
| *PKD2* |  |  |  |  |  |  |  |
| IVS4 | c.1095-32A>G |  | IVS Silent | P | PM |  | Novel |
| EX4 | c.1354 A>G | p.Ile452Val | Substitution | P | PM | rs1801612 | PD |
| EX6 | c.1546 G>T | p.Val516Leu | Substitution | P | PM | rs143581690 | NCBI DbSNP |

PD, previously described in other studies, could be seen in the Autosomal Dominant Polycystic Kidney Disease: Mutation Database (PKDB); P, detected from patients; UF, detected from unaffected family members; NC, detected from normal controls; PM, polymorphism; PPM, probable polymorphism; p.(=), synonymous change at protein level.
